# Speech pathologists’ experiences with stroke clinical practice guidelines and the barriers and facilitators influencing their use: a national descriptive study

**DOI:** 10.1186/1472-6963-14-110

**Published:** 2014-03-06

**Authors:** Kathleen A Hadely, Emma Power, Robyn O’Halloran

**Affiliations:** 1 Discipline of Speech Pathology, Faculty of Health Sciences, The University of Sydney, 75 East Street, Lidcombe NSW 2141, Australia; 2 Centre for Clinical Research Excellence in Aphasia Rehabilitation, Brisbane, Australia; 3 Department of Human Communication Sciences, La Trobe University, PO Box 199, Bendigo VIC 3552, Australia

**Keywords:** Speech pathologist, Stroke, Rehabilitation, Clinical practice guideline, Knowledge translation, Knowledge to action framework, Barriers, Facilitators, Implementation

## Abstract

**Background:**

Communication and swallowing disorders are a common consequence of stroke. Clinical practice guidelines (CPGs) have been created to assist health professionals to put research evidence into clinical practice and can improve stroke care outcomes. However, CPGs are often not successfully implemented in clinical practice and research is needed to explore the factors that influence speech pathologists’ implementation of stroke CPGs. This study aimed to describe speech pathologists’ experiences and current use of guidelines, and to identify what factors influence speech pathologists’ implementation of stroke CPGs.

**Methods:**

Speech pathologists working in stroke rehabilitation who had used a stroke CPG were invited to complete a 39-item online survey. Content analysis and descriptive and inferential statistics were used to analyse the data.

**Results:**

320 participants from all states and territories of Australia were surveyed. Almost all speech pathologists had used a stroke CPG and had found the guideline “somewhat useful” or “very useful”. Factors that speech pathologists perceived influenced CPG implementation included the: (a) guideline itself, (b) work environment, (c) aspects related to the speech pathologist themselves, (d) patient characteristics, and (e) types of implementation strategies provided.

**Conclusions:**

There are many different factors that can influence speech pathologists’ implementation of CPGs. The factors that influenced the implementation of CPGs can be understood in terms of knowledge creation and implementation frameworks. Speech pathologists should continue to adapt the stroke CPG to their local work environment and evaluate their use. To enhance guideline implementation, they may benefit from a combination of educational meetings and resources, outreach visits, support from senior colleagues, and audit and feedback strategies.

## Background

### The impact of a stroke

Globally, around 15 million people suffer a stroke each year [1]. Approximately one third of these people acquire permanent disability [1]. Many people experience the sudden onset of a communication disability following a stroke. For example, it is estimated that 35% of people acquire aphasia [2], 58% of people present with dysarthria [3], and approximately 25-77% of individuals have a cognitive impairment that may impact on communication [4] post stroke. In addition, between 64%-78% of people experience a swallowing disability [5] after a stroke. These disabilities can have a devastating impact on a person’s life [6,7].

Every year an enormous amount of new research is published on the assessment and management of communication and swallowing disorders post stroke. For patients to benefit from this research, speech pathologists need to regularly search, appraise, and integrate this new knowledge into their clinical practice. However, this can be difficult to do due to a lack of expertise in analysing and appraising research evidence combined with the demands of large and varied caseloads [8,9].

### The knowledge-to-action process

**Figure 1:**
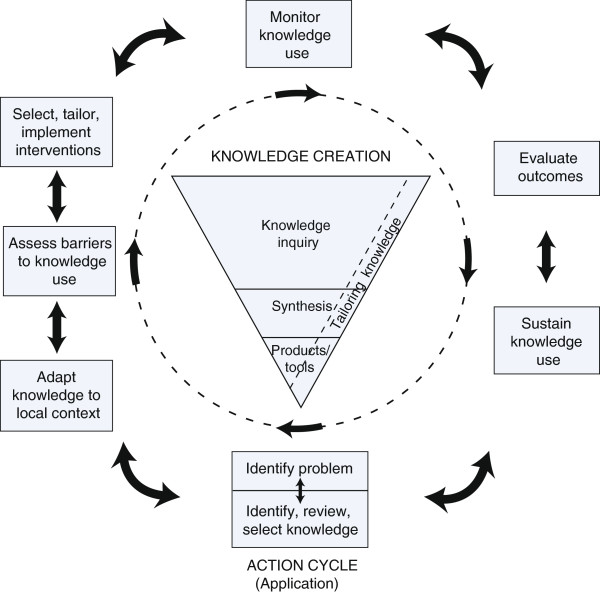
**The knowledge-to-action process framework **[11]**.** Reprinted from Straus SE, Tetroe J, & Graham, Defining knowledge translation, Canadian Medical Association Journal (2009, 181, 165-168). ©Canadian Medical Association (2009). This work is protected by copyright and the making of this copy was with the permission of the Canadian Medical Association Journal (http://www.cmaj.ca) and Access Copyright. Any alteration of its content or further copying in any form whatsoever is strictly prohibited unless otherwise permitted by law.

The Knowledge-to-Action Process (KTA) [10] is one framework based on planned-action theories that proposes that there are critical processes that enable knowledge, such as research evidence, to be successfully and continually implemented into clinical practice (see Figure 1). The KTA framework consists of two components: the ‘Knowledge Creation’ component and the ‘Action Cycle’ component.

The ‘Knowledge Creation’ component states that knowledge needs to be synthesised, refined, and tailored into a tool, to increase its practicality [11]. The ‘Action Cycle’ describes eight processes that may need to occur for the successful implementation of the knowledge (or tool) into clinical practice. These eight processes include (a) identifying a problem, that is a knowledge to practice gap; (b) identifying, reviewing, and selecting knowledge relevant to the problem; (c) adapting the knowledge to the clinical setting, (d) assessing barriers to using the knowledge, (e) selecting, tailoring, and implementing interventions to promote use of the knowledge; (f) monitoring use of the knowledge, (g) evaluating outcomes of using the knowledge, and (h) sustaining use of the knowledge [10].

A clinical practice guideline (CPG) is one type of knowledge creation tool. It is defined as “statements that include recommendations intended to optimise patient care that are informed by a systematic review of evidence and an assessment of the benefits and harms of alternative care options” [12]. Thus, CPGs can assist health professionals to implement critically appraised evidence into clinical practice more efficiently and effectively. The implementation of stroke CPG recommendations is also associated with better post stroke recovery outcomes [13]. Therefore, the successful implementation of stroke CPGs can help benefit patients who have had a stroke by improving the level of stroke care provided by their health professionals.

However stroke CPGs are not always implemented and thus may have little impact on patient outcomes. An Australian audit of 111 hospitals by the National Stroke Foundation found many practice gaps between specific recommendations in the *Clinical Guidelines for Stroke Management 2010* and clinical practice, and concluded that there had been minimal improvement since the first audit in 2008 [14]. Similarly, the National Sentinel Stroke Audit audited 200 acute hospitals across England, Wales, and Northern Ireland on the *National Clinical Guideline for Stroke Third Edition*[15]. Although compliance had increased for all standards between 2004 and 2010, there were still gaps between what was recommended in the stroke CPG and clinical practice. In 2012, The Scottish Stroke Care Audit audited all hospitals that managed acute stroke in Scotland [16]. This audit also found that some stroke recommendations were poorly implemented in hospitals. Therefore, despite clear recommendations for health professionals, there is a common issue globally that CPGs are not consistently and fully implemented into clinical practice.

### Factors influencing the use of clinical practice guidelines

Considerable research has focused on the factors that influence health professionals to implement CPGs. A recent systematic meta-review has summarised the results of 12 systematic reviews on the factors that influence guideline implementation [17]. This meta-review concluded that factors that influence implementation could be categorised into five areas: (a) the guideline itself, for example, guidelines that did not require specific resources were easier to implement; (b) the health professional, where less experienced health professionals are more likely to implement guidelines than more experienced health professionals; (c) patient characteristics, where patients with co-morbidities resulted in less guideline adherence by their health professionals; (d) the work environment, for example, limited resources and negative attitudes from colleagues lead to less CPG adherence; and (e) the type of implementation strategy used, such as multifaceted intervention (using two or more strategies) being more effective in implementing CPGs than using one strategy only.

Systematic reviews have also examined the effectiveness of the type of strategy used to improve CPG implementation. A large-scale systematic review in 2004 evaluated the effectiveness of strategies from 235 studies [18]. This review reported that providing reminders was the most frequent strategy evaluated and was moderately effective in improving guideline implementation. Educational outreach was the next most common strategy evaluated and had a modest effect in increasing CPG adherence. Multifaceted interventions were found to be no more effective than single strategies in improving implementation [18]. However another systematic review in 2008 [19], reported that multifaceted interventions, interactive education, and clinical reminder systems were effective. Finally, a systematic review by Medves and colleagues [20] on the strategies that enhance dissemination and implementation of clinical guidelines in healthcare teams, reported that the two most common strategies used to implement guidelines recommendations were educational materials and educational meetings; whilst the most effective strategies were reminders, audit and feedback, and opinion leaders. Although there is evidence on the variety of factors that influence guideline implementation by health professionals, the majority of these systematic reviews have focused on physicians, then nurses, with a small number of studies investigating other health professionals. The factors that influence physicians and nurses to implement CPGs may not be the same as those that influence allied health professionals.

One systematic review on the effectiveness of strategies to disseminate and implement guidelines for allied health professionals has been conducted by Hakkennes and Dodd [21]. This study concluded that there was no evidence to support a set guideline implementation strategy for allied health professionals. The review also found that multifaceted interventions were no more effective than single intervention strategies. The authors suggested that implementation strategies are only effective if they address identified local barriers to change.

Since Hakkennes and Dodd’s [21] systematic review, other studies on the effectiveness of strategies to implement CPGs in allied health professionals has been conducted. Three recent studies [22-24] have examined the factors that influenced allied health professionals’ use of CPGs. These factors can be categorised into the five broad areas identified previously i.e. the guideline itself, the health professional, patient characteristics, the work environment, and the type of implementation strategy. However, there were several factors observed in allied health professionals that were not reported in research on physicians. One example related to the work environment, where allied health professionals who did not work in an interdisciplinary team were found to adhere less to CPGs [22]. Another example related to patient characteristics, where patients who were not motivated to become less disabled made it difficult for occupational therapists to apply guidelines [24]. These factors were not identified in the systematic reviews on physicians. Overall, these studies revealed that the factors that influence allied health professionals may differ from physicians. A list of factors that influence the implementation of CPGs for allied health professionals are listed in Table 1.

**Table 1:** Key factors that influence the implementation of clinical practice guidelines in allied health professionals

**Area**	**Factors**	**Professionals studied**	**References**
(a) Aspects relating to the guideline itself	Clarity of recommendations	Physiotherapists, occupational therapists	[23,24]
	Applicability to clients		
	Amount of detail provided		
	Allowed the health professional to draw their own conclusions		
	Allowed the health professional to take the client’s preferences into account		
(b) Characteristics of the health professional	Desire to maintain accountability	Physiotherapists, occupational therapists, nurses, managers	[22,24]
	Willingness to change practice		
	Agreement with the guidelines		
	Level of knowledge		
	Level of skill		
(c) Patient characteristics	Severity of the patient	Physiotherapists, occupational therapists, nurses, managers	[22,24]
	Patient motivation		
	Patient expectations		
(d) Work environment	Time availability	Physiotherapists, occupational therapists, nurses, managers	[22-24]
	Staff availability		
	Training and education		
	Workplace policies		
	Team collaboration		
	Access to other professionals		
	Colleagues		
(e) Implementation strategies	Multifaceted interventions were no more effective than using one strategy only. There is no clear evidence to support a set guideline implementation strategy for allied health professionals.	Pharmacists (8 studies), physiotherapists (3 studies), dietitians (2 studies), and speech pathologists (1 study)	[21]

Factors that influence the implementation of CPGs can also vary across different allied health professionals. A recent study indicated that factors influencing CPG use for physicians, occupational therapists, and physiotherapists differed for each occupation [25]. For example, barriers related to the workplace hindered the use of CPGs for occupational therapists, whereas, issues related to the workplace were rarely identified by physiotherapists. Therefore generalisation from these more commonly researched allied professions to another profession may not be valid. To date, no study has included speech pathologists, and thus the factors that influence speech pathologists’ implementation of guidelines in unknown. Additionally, the reviews conducted thus far have only focused on two aspects of the KTA cycle i.e. (1) the identification of barriers and facilitators that improve guideline adherence and (2) examining effective implementation strategies to improve CPG dissemination. As most evidence has mainly focused on these two aspects of the KTA cycle, little is known about whether other aspects of the KTA cycle influence implementation. This difficulty has recently been documented in relation to occupational physicians use of weight gain prevention CPGs [26]. None of the seven occupational physicians in this study continued to use the CPG guidelines six months after they received tailored implementation strategies. Therefore, issues surrounding KTA components such as monitoring knowledge use and sustaining knowledge use are rarely addressed.

In summary, little is known about the factors that influence the implementation of guidelines in speech pathology. There is also limited research on how to sustain the use of knowledge such as CPGs in clinical practice. Understanding how speech pathologists currently use CPGs as well as considering processes of the KTA framework may give insight into the specific factors that influence speech pathologists to use and sustain the use of CPGs. The aims of this study are to (a) describe speech pathologists’ experiences and current use of stroke CPGs and (b) identify what factors influence speech pathologists to use and maintain the use of stroke CPGs.

## Method

As this study aimed to describe the experiences and use of stroke CPGs across a broad cross section of speech pathologists, a survey method was used.

### Participants

Qualified speech pathologists in Australia were invited to participate in this study if they: (a) had worked with at least one client in the last five years who had a stroke and (b) had used a stroke CPG.

### Design of survey

A 39-item online survey was constructed using the survey design website SurveyMonkey® [27]. As there are currently few reliable and valid tools for assessing barriers and facilitators to guideline implementation [28], survey questions were thus developed from (a) research evidence on CPG implementation, (b) reference to a theoretical framework [10], and (c) consultation with a project reference group. The group consisted of a representative from the National Stroke Foundation, a clinical researcher, and a speech pathology manager, who were all experienced in the development of CPGs. The survey included a variety of response formats, including dichotomous choice (yes/no); multiple choice, and open ended text box options. Most questions (19/39) allowed participants to write additional responses that were not pre-classified.

The study reference group was invited to provide feedback on the survey content, wording, format, and length. This ensured that all survey questions were relevant and that survey completion was feasible for participants. Following this consultation, filter questions such as “Has the work environment helped you to continue to use the stroke clinical practice guideline?”, were inserted to reduce response requirements and the length of time taken to complete the survey [29]. The survey was then piloted with three speech pathologists and three speech pathology students resulting in minor changes to the wording of some items.

This process resulted in a 39 item survey covering six domains of: (a) demographics (Q1-13), (b) initial experiences with and impressions of stroke CPGs (Q14-23), (c) facilitators to using stroke CPGs (Q24-28), (d) barriers to using stroke CPGs (Q29-33), (e) evaluating outcomes of stroke CPGs (Q34-38), and (f) any additional comments regarding stroke CPGs (Q39). The survey questions are provided in Additional file 1: Table S1.

### Recruitment and data collection

The online survey was opened for eight weeks between 5^th^ April, 2012 and 31^st^ May, 2012 and a survey reminder was emailed 10 days prior to closing date to maximise participation [30]. The distribution of the survey was extensive and targeted. Speech pathologists who worked in stroke in both public and private settings and in metropolitan, rural, and remote areas in Australia were targeted using a variety of networks. Specifically, these networks included speech pathologists from all parts of Australia (electronic member newsletter of Speech Pathology Australia); speech pathologists working in aphasia (electronic member newsletter of the Centre for Clinical Research Excellence in Aphasia rehabilitation), speech pathologists in adult rehabilitation (special interest group called SPECS), private speech pathologists (private practitioner networks), and speech pathologists working in adult stroke (NSW Statewide Stroke Services’ *“SSNSW Stroke Distribution List no. 1 & 2”* and the National Stroke Foundation’s “*National Stroke Foundation Health professional database”*). Ethical clearance was approved by the University of Queensland and University of Sydney Human Ethics Committees.

### Data preparation and analysis

#### Data screening

Data from SurveyMonkey® was exported into a Microsoft Office Excel 2007^©^ spreadsheet. Each participant was assigned a unique numerical ID by SurveyMonkey® to maintain anonymity. The data was first screened to identify any missing values and to ensure valid responses. Participants who indicated in Question 1 that in the last five years, they had not worked with a client who has had a stroke, were excluded from further analysis. Additionally, participants who reported in Question 15 that they had not used a stroke CPG were also removed from further analysis.

#### Data analysis

A summary of each question including a table of absolute frequencies, proportions, and graphs was completed to allow for visual inspection of the data. Descriptive statistics were used to analyse the data. Confidence intervals (Exact 95% C.I. [Mid-P]) were calculated using WINPEPI [31] to determine if there were any differences between (a) speech pathologists who had and had not used stroke CPGs and (b) demographic proportions of the current study compared with proportions from the most recent Speech Pathology Australia member workforce survey (data provided by Speech Pathology Australia [32]). To analyse the open ended responses, a simple content analysis based on Elo and Kyngäs [33] and Hsieh and Shannon [34] was performed (i.e. open-coding, coding sheets, and abstraction). Each open ended response was read and given an open code that summarised the content of the response (sub-category). Similar open codes were then grouped together, to form main categories. A title was given to each main category and a brief description that explained each category was provided (abstraction). Finally, similar main categories were grouped together to form major categories, and a final heading that described the content/characteristic of each major category was provided. For example, the question “*How have stroke clinical practice guidelines helped improve the care you provide?”* elicited 190 open ended responses. One response was: *“the stroke clinical practice guidelines help improve care by providing evidence to assist in advocating for optimal care for patients”*. This response was given the open code of: “*Provides evidence to help advocate for optimal care for patients*” (sub-category). This response and other similar responses were grouped together to form a main category titled “Advocacy”. A description that explained the “advocacy” category was created (abstraction): *“Advocate for patients e.g. aphasia friendly materials, clients’ needs, early intervention, more communication management”*. Finally, the group “Advocacy” was placed with other similar categories (e.g. the guidelines helped changed practice, prioritise services, develop policies and procedures) to form a major category. This major category was given the title of *“Guidelines help to develop or improve services”* and was one of the four main categories identified for this question through content analysis. The coded responses were peer checked by the third author (RO) to enhance the credibility of the results [35].

## Results

**Figure 2:**
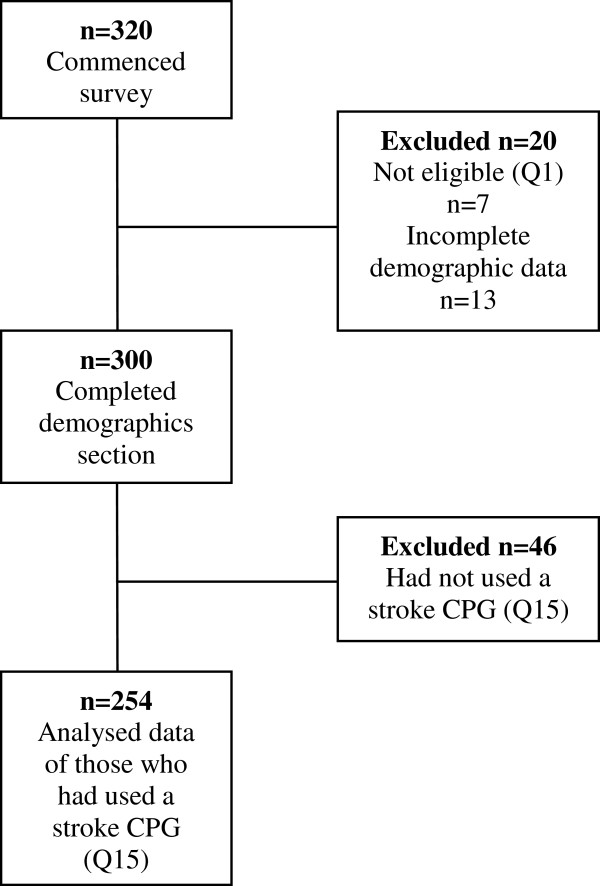
Flowchart depicting participant flow from the commencement of the study through to final participant sample.

A total of 320 speech pathologists commenced the survey. Twenty participants were excluded from further analysis because they had either not seen a patient with a stroke in the last five years (n = 7), or they dropped out of the survey and did not complete the demographic questions (n = 13). This left 300 participants who completed the demographic section. At this stage a further 46 participants were excluded as they had not used a stroke CPG (Q15). Therefore, a total of 254 respondents completed the remainder of the survey. A flow diagram describing the participants in this study is provided in Figure 2.

### Demographic profile of eligible participants (n = 254) and demographic characteristics that influence stroke clinical practice guideline implementation (Questions [Q] 2-15)

The demographic profile of the speech pathologists who completed this survey is provided in Table 2. A comparison of the proportions of speech pathologists in this sample with the most recent Speech Pathology Australia member workforce survey [32] found few significant differences. The current study did however have a significantly larger proportion of those working in the rural setting (30.4%), compared to the 2003 workforce survey (13.8%); demonstrated by a lack of confidence interval overlap (95% CIs [0.2538 to 0.3572] and [0.113 to 0.166] respectively).

**Table 2:** Participant demographics (n = 254 unless otherwise stated)

**Variables**	**N**	**%**
Gender		
Male	8	3.5%
Female	246	96.5%
Age		
20-30 years	123	48.2%
31-40 years	76	30.2%
41-50 years	32	12.5%
51-60 years	19	7.5%
61-64 years	4	1.6%
65 + years	0	0.0%
No. of years since graduation		
Less than 5 years	83	35.5%
5-10 years	73	29.0%
10-20 years	57	22.4%
More than 20 years	41	16.1%
Highest level of academic achievement		
Bachelor	148	58.4%
Honours	42	16.5%
Post graduate certificate/diploma	9	3.5%
Masters	46	18.0%
PhD	9	3.5%
State/Territory of work environment		
Australian capital territory	2	0.8%
New South Wales	89	35.0%
Northern territory	3	1.2%
Queensland	70	27.6%
South Australia	16	6.3%
Tasmania	8	3.1%
Victoria	48	18.9%
Western Australia	18	7.1%
Work region		
Metropolitan	175	68.6%
Rural	75	29.8%
Remote	4	1.6%
Work environment		
Government	227	89.0%
Non-profit organisation	8	3.5%
Private practice	9	3.5%
University	5	2.0%
Other	5	2.0%
Clinical continuum of care setting (n = 106)		
Acute setting	27	25.5%
Inpatient setting	30	28.3%
Outpatient setting	12	11.3%
Community setting	15	14.2%
Residential care setting	1	0.9%
Combination of above	21	19.8%
Multidisciplinary team		
Members of a multidisciplinary team	245	96.5%
Not members of a multidisciplinary team	9	3.5%
Dedicated stroke unit team		
Members of a multidisciplinary team who were part of a dedicated stroke unit team	99	40.2%
Members of a multidisciplinary team who were not part of a dedicated stroke unit team	146	59.8%
Years working with neurogenic communication disorders		
1-5 years	115	45.5%
6-10 years	63	24.7%
11-15 years	32	12.5%
16-20 years	22	8.6%
More than 20 years	22	8.6%
Approximate percentage of caseload that contains people who have had a stroke		
Less than 5%	15	5.9%
5%	10	3.9%
10%	17	6.7%
30%	49	19.3%
50%	63	24.8%
75%	84	33.1%
100%	16	6.3%

To determine whether the demographic characteristics of those who used stroke CPGs (n = 254) were different from those who had not (n = 46), confidence intervals were compared across the two groups. A significantly greater proportion of speech pathologists who had not used a stroke CPG worked in private practice, had 1-5 years experience, or did not work in a multidisciplinary team.

### Speech pathologists and stroke clinical practice guidelines (n = 254)

The following results describe the responses of the 254 respondents who completed the remainder of the survey. These results report on (a) speech pathologists’ initial experiences and current use of stroke CPGs and (b) factors that influence speech pathologists to use and maintain the use of stroke clinical practice guidelines.

The total number of responses differs for each question as participants were allowed to tick more than one answer on some items, filter questions were used, and some questions were not answered. The total number of responses is shown in the graphs provided.

#### Speech pathologists’ initial experiences and current use of stroke clinical practice guidelines

This section describes the usefulness of stroke CPGs and the predominant stroke CPG used by speech pathologists, their awareness of stroke CPGs, the main uses of stroke CPGs, how stroke CPGs have helped improve the care they provide to patients, why stroke CPGs have not helped improve the care provided to patients, and the strategies used to evaluate the implementation of stroke CPGs.

#### Usefulness of stroke clinical practice guidelines and the predominant stroke clinical practice guideline used (Q17-18)

Most respondents reported that the stroke CPGs were “somewhat useful” (63.3%) or “very useful” (34.4%). Six participants reported that stroke CPGs were “not really useful” (2.0%) or “not useful at all” (0.40%). The *National Stroke Foundation: Clinical Guidelines for Stroke Management 2010* was the most commonly used guideline (88.6%). Approximately half of the participants (53.5%) had also used the 2007 National Stroke Foundation edition. Nearly one-fifth (18.5%) of respondents reported using a stroke CPG that was used and/or created by their own workplace. Thirteen participants provided examples of these documents that included international guidelines from Canada *(Canadian stroke guidelines)*, New Zealand *(New Zealand Clinical Guidelines for Stroke Management 2010)*, Scotland (*SIGN 2008*), United Kingdom (*RCSLT Communicating Quality),* and the United States *(Va Clinical Guidelines)*; and local guidelines including the *NSF: Concise Guidelines–Speech Pathology–Online* and the *Statewide Stroke Clinical Network: Pathway for Stroke Rehabilitation–A Best Practice Guide for Stroke Rehabilitation Services in South Australia*.

#### How speech pathologists became aware of stroke clinical practice guidelines (Q14, 16, 19)

**Figure 3:**
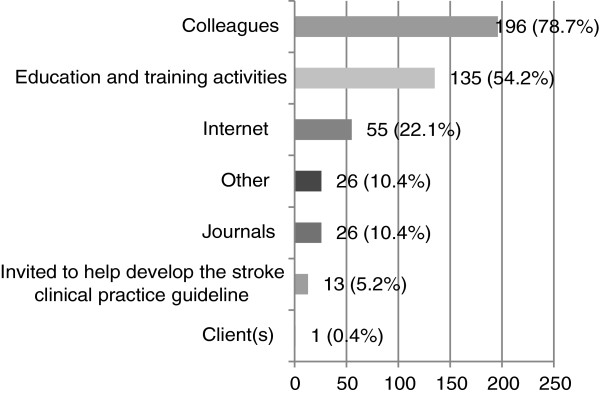
**How participants became aware of stroke CPGs (n = 249).** (Participants were able to choose more than one answer).

Almost all speech pathologists (95.0%) had heard of stroke clinical CPGs and had read the stroke CPG (92.9%). Most speech pathologists became aware of stroke CPGs through colleagues (78.7%) and education and training activities (54.2%). Further information about how speech pathologists became aware of CPGs is provided in Figure 3.

#### The main uses of stroke clinical practice guidelines (Q37)

**Figure 4:**
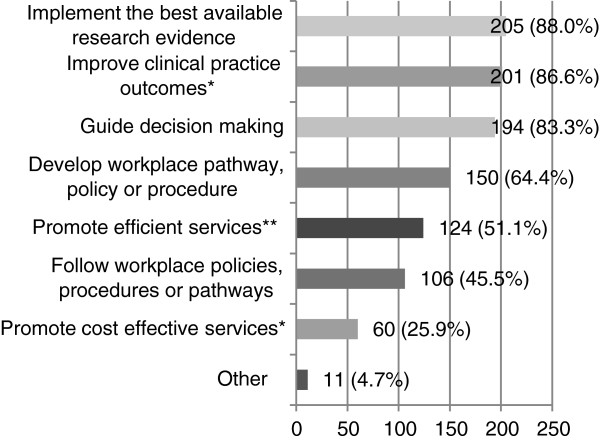
**The uses of stroke CPGs (n = 233 unless otherwise specified).** Participants were asked to indicate the main reasons why they used stroke CPGs. *n = 232. **n = 229.

Speech pathologists mainly used stroke CPGs to implement the best available research evidence (88.0%), improve clinical practice outcomes (86.6%), and guide decision-making (83.3%) (see Figure 4). A total of 150 respondents (64.4%) also indicated that the CPGs were mainly used to develop policies, procedures, or pathways. Other reasons to use the guideline included advocating for clients and services, educating patients and carers, highlighting the need for more research, and to ensure that speech pathologists were providing the recommended care. For example, one speech pathologist reported that the guidelines “*Provided overall support for management of stroke which can be used to justify therapy programs (e.g. highlight the need for intensive aphasia therapy to a consultant who is promoting early discharge of a mobile patient with severe aphasia)”*.

#### How stroke clinical practice guidelines have helped improve the care speech pathologists provide (Q34, 36)

The stroke CPGs were reported to have helped 190 (80.5%) participants improve the care they provided. Using content analysis, the ways stroke CPGs helped to improve care could be divided into four categories.

a. *Stroke clinical practice guidelines provide a standard you can trust.* Speech pathologists reported that the guidelines had provided a framework for services, guided them and others regarding what to do, made stroke services more consistent, and were something to refer to. For example, one respondent wrote *“They provide a framework for appropriate stroke management and to ensure the patient/client is receiving therapy based on current best practice”*.

b. *Guidelines help to develop or improve services.* Respondents also stated that guidelines helped them advocate for clients or services, encouraged client centred or multidisciplinary care, helped to prioritise referrals, changed practice, supported the development of policies and practices, and helped improve goal setting. For example, one respondent stated that the guidelines had led to *“Less (patients with) aspiration pneumonia and less patients inappropriately being kept nil by mouth”*. Another respondent wrote that the guidelines had *“Enabled speech pathologists to develop a nurse screening training program for swallowing screening which has allowed speech pathologists to spend more time on assessment and management of patients’ communication”*.

c. *Stroke clinical practice guidelines enhance a sense of self as a speech pathologist.* Speech pathologists reported that the guidelines made them feel more confident in the services they were providing, gave them confidence in delivering evidence-based practice, and had helped them with health professional decision-making. For example, one participant wrote that the guidelines had *“Enhanced my confidence relating to the amount/intensity of therapy I provide, the specific areas of impairment I target, and the specific therapy approaches I use”*.

d. *Stroke clinical practice guidelines support engagement in research evidence.* Participants also indicated that the guidelines had made it easier for them to access the literature and also encouraged them to read more evidence, for example, *“It is an easy way to keep up to date with a broad range of clinical research relating to stroke care”* and another commented that the guidelines *“encouraged further reading and review of literature”.*

#### Why stroke clinical practice guidelines have not helped improve the care speech pathologists provide (Q34, 35)

Forty-six respondents (19.5%) indicated that the stroke CPGs had not helped improve the care they provided. Responses were categorised into four main groups.

a. *The stroke clinical practice guideline is not adding anything new to my practice.* Twelve respondents reported that the guideline did not add anything new to their practice or knowledge base as *“I was already following most of the practical care based recommendations, so I don’t think the care I provide is all that different”*.

b. *The stroke clinical practice guideline has limitations for my practice.* Three speech pathologists reported that the guideline had limitations due to a lack of detail for long term chronic management of stroke as the CPGs *“don’t specifically mention long term chronic management of stroke symptoms which is the area where I work”* and for being too broad to provide specific direction for client management, evidenced in the statement, *“I feel that many of the recommendations are quite broad (i.e. use a screening tool for aphasia, provide information, talk to caregivers etc)”.* Stroke guidelines were also limited because they were not *“updated quickly enough to support progressive practice”*.

c. *Organisational support is needed to implement the stroke clinical practice guideline.* Participants indicated that a lack of organisational support in relation to resources, education, leadership, and guideline enforcement also meant guidelines did not help improve their practice. For example clinicians reported that they *“… haven't got the time and resources to implement the guidelines sufficiently to improve patient care”* and that budgetary and staffing constraints reduced their ability to meet the CPG recommendations. For example, *“poor staffing of speech pathologists in our local rehabilitation services means that the Area is no way near meeting any of the guidelines*”. For one respondent working in the private sector, the challenge of finding time to implement the guidelines and keep up to date was conceptualised in terms of difficulty balancing *“billable versus non-billable time*”. A lack of education for speech pathologists and the need for “*further explanation and training”* was cited to be an important element in *“… maximising stroke care provided within workplace”.* This extended to other professions and services as one respondent commented: *“Education is not provided to frontline emergency staff. Referral to Allied health via the stroke pathway is not consistently implemented*”. Finally, a lack of leadership and guideline enforcement at a service level reduced the ability to maximise patient outcomes, particularly in the acute setting: *“The guidelines are not translated successfully in the acute setting in terms of stroke management pathways within the hospital. Adherence to the stroke pathways in the acute setting is not audited”*.

d. *Implementing stroke clinical practice guidelines is not a priority.* One respondent also reported that the implementation of the guideline was not a priority due to competing policies indicating there are *“Many other policies and guidelines to implement at (the) workplace”*.

#### Strategies used to evaluate implementation of stroke clinical practice guidelines (Q38)

The most common strategies used to evaluate guideline adherence included the National Stroke Foundation audit (45.3%), other workplace audits (34.9%), and the use of quality indicators (32.8%). One-third of participants indicated that no CPG evaluation had taken place (30.2%).

### Factors that influence speech pathologists to use and maintain the use of stroke clinical practice guidelines

The following results describe the barriers and facilitators that influenced speech pathologists’ implementation of stroke CPGs.

#### Barriers that hindered the continued use of stroke clinical practice guidelines

Speech pathologists described four broad barriers that hindered the continued implementation of stroke CPGs in clinical practice. These barriers included the work environment, the guideline itself, barriers relating to the speech pathologists themselves, and patient characteristics.

##### Work environment as a barrier (Q31-32)

**Figure 5:**
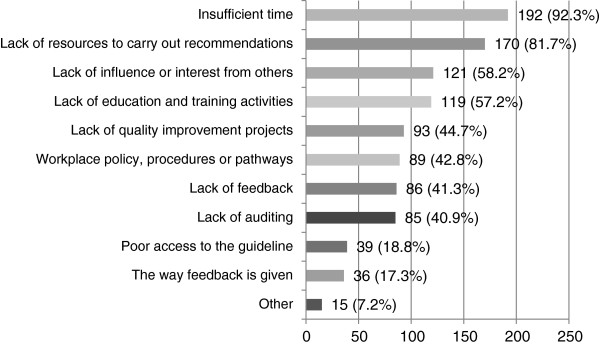
**How the work environment hindered the continued use of stroke CPGs (n = 208).** Participants who reported that the work environment hindered their use of stroke CPGs were asked to specifically indicate how.

The work environment was the main barrier to CPG implementation for the majority of respondents (87.8%), and insufficient time (92.3%) and a lack of resources to carry out recommendations (81.7%) were the two top reasons cited (see Figure 5). This was followed by a lack of interest or influence from others (e.g. colleagues) (58.2%) and education activities (57.2%). For example, *“Insufficient local resources create huge barriers to complying with the guidelines, whether it’s staffing, insufficient treatment spaces etc … if the consultant doctors are not on-board with the guidelines, it can be difficult to advocate for the person’s length of admission that allows them access to the duration and intensity of multidisciplinary input due to bed-pressure”.*

##### Aspects of the stroke clinical practice guideline itself as a barrier (Q29-30)

**Figure 6:**
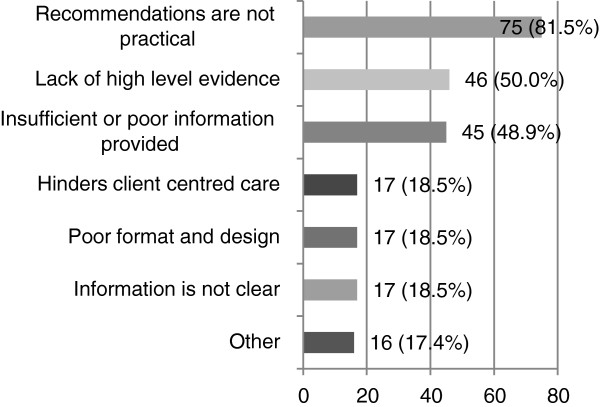
**How aspects of the guideline itself hindered the continued use of stroke CPGs (n = 92).** Participants who reported that aspects of the guideline itself hindered their use of stroke CPGs were asked to specifically indicate how.

Ninety-three participants (31.9%) indicated that the guideline itself was a barrier to implementing the recommendations. A total of 81.5% reported that the recommendations were not practical (see Figure 6). Half of the respondents also indicated that a lack of high-level evidence (50.0%) and insufficient or poor information (48.9%) hindered CPG use. One respondent wrote *“(The guidelines) are not detailed enough and effectively represent a stand alone document, rather than a package or suite of tools to put them into clinical practice. They’re not dynamic in a web sense, but static on a page. They don’t open up and show you resources or examples of treatments. They are not accompanied by clinical resources that might be standardised to use in implementation. Talk about reinventing the wheel. It’s just too hard, so we haven’t got there”.*

##### Barriers relating to the speech pathologist themselves (Q33)

**Figure 7:**
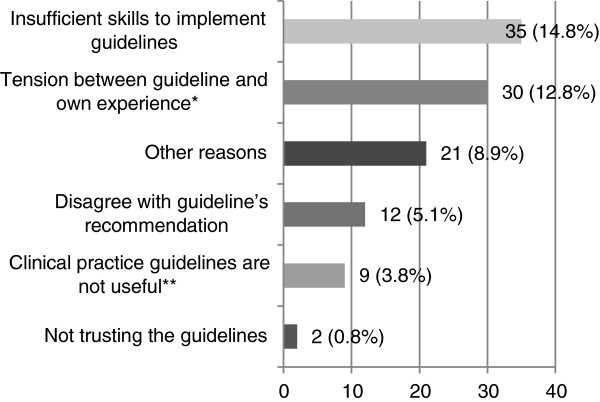
**Aspects related to the participant that hindered the continued use stroke CPGs (n = 237 unless specified).** *n = 235. **n = 236.

The main reasons that made speech pathologists feel reluctant to use the stroke CPG were “insufficient skills to implement guidelines” (14.8%) and a “tension between guideline and own experience” (12.8%) (see Figure 7). Fifteen participants provided comments that further explained these factors. For example, *“Some of the dysarthria recommendations are not what I would routinely use for stroke…”* and another explained that *“Sometimes there is a tension between the guideline and my own experience but only in some cases”*.

##### Barriers relating to patients (Q33)

Two speech pathologists also commented on patient characteristics that hindered their use of guidelines in this question. This included patients who were at *“*… *the more severe end of the stroke spectrum, often with multiple and complex co-morbidities”* and *“… severe aphasics as they aren’t usually able to access the same intensity likely resulting in poorer outcomes”*.

#### Facilitators that influenced the continued use of stroke clinical practice guidelines

This section will describe the five broad facilitators that influenced speech pathologists to continue to use stroke CPGs. These facilitators included the work environment, aspects of the guideline itself, facilitators relating to the speech pathologists themselves, patient characteristics, and the type of guideline implementation strategy used.

##### Work environment as a facilitator (Q26-27)

**Figure 8:**
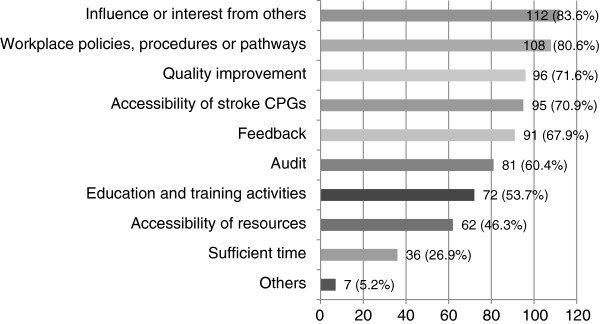
**How the work environment facilitated the continued use of stroke CPGs (n = 134).** Participants who reported that the work environment facilitated their use of stroke CPGs were asked to specifically indicate how.

Just over half (55.8%) of respondents indicated that the work environment had facilitated continued use of the stroke CPG. Influence or interest from others (e.g. colleagues) (83.6%) and workplace policies, procedures, or pathways (80.6%) were the top two responses (see Figure 8). Additionally, around three-quarters of participants reported that quality improvement projects (71.6%) and accessibility of stroke CPGs (70.9%) had also helped facilitate CPG use. Nearly two-thirds of respondents indicated feedback (67.9%) and audit (60.4%) was a facilitator for continued use of the guideline. Other facilitators included staff training, support and auditing from a rehabilitation working party, and making the stroke CPG a high priority by the manager.

##### Aspects of the stroke clinical practice guideline itself as a facilitator (Q24-25)

**Figure 9:**
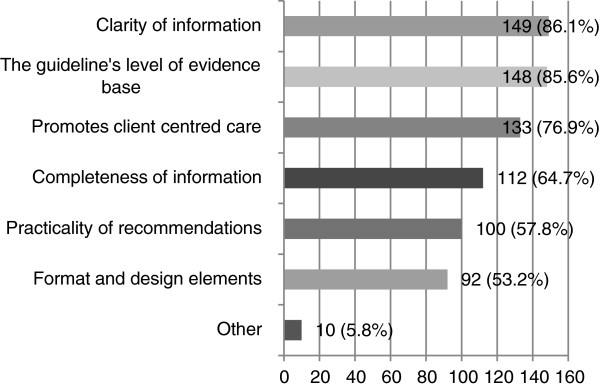
**How aspects of the guideline itself facilitated the continued use of stroke CPGs (n = 173).** Participants who reported that aspects of the guideline itself facilitated their use of stroke CPGs were asked to specifically indicate how.

Characteristics of the guideline itself helped 176 respondents (72%) continue to use it. The top three aspects were the clarity of information in the guideline (86.1%), the guideline’s level of evidence base (85.6%), and the guideline promoting client centred care (76.9%) (see Figure 9). Ten participants provided answers in the ‘other’ open-ended text box. Five respondents reported facilitators to continued use of the stroke CPG such as the guideline’s comprehensive reference list, the guideline being easy to read and navigate, tools in the guideline (e.g. algorithms, flowcharts, and summary documents); and the information in the guideline being a “*great overview…”*.

##### Facilitators relating to the speech pathologists themselves (Q28)

Almost all participants indicated that a desire to implement evidence-based practice has helped motivate them to use the guidelines (97.9%) e.g. *“My aim is to do the best for my client with assessment and therapy and aim to return to the best function possible based on evidence…”*. This was followed by agreeing with the recommendations in the stroke CPG (83.3%), having the skills to use the guideline (81.7%), and the recommendations in the guideline being similar to their own experience (74.6%).

##### Patient characteristics as a facilitator (Q36-37)

Patients who had severe aphasia influenced speech pathologists to use stroke CPGs as a tool to advocate for more intensive therapy and longer stay. For example, one speech pathologist commented that they *“highlight(ed) the need for intensive aphasia therapy to a consultant who is promoting early discharge of a mobile patient with severe aphasia”*. Another reported that they used the guidelines when *“… advocating for patients particularly those who are doing well physically, but whom have severe aphasia”*.

##### Implementation strategies as a facilitator (Q20-23)

**Figure 10:**
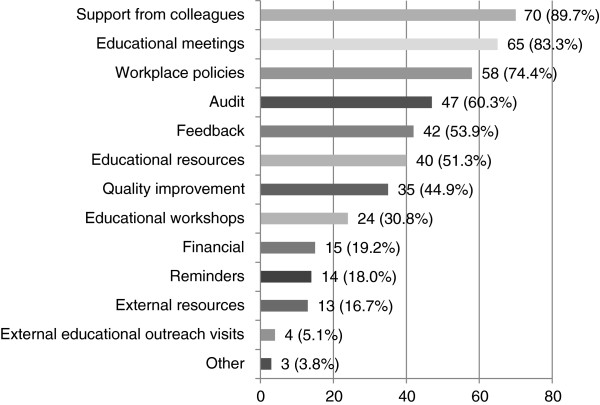
**Implementation strategies provided to participants after dissemination of stroke CPGs (n = 78).** Participants were asked to indicate the type of guideline implementation strategy(s) provided after receiving the stroke CPG.

Implementation strategies to assist the dissemination of stroke CPGs were provided to 80 (32.3%) of the 248 speech pathologists (see Figure 10). The most frequent strategies provided were support from colleagues (89.7%), educational meetings (83.3%), and/or workplace policies (74.4%). All but one participant received two or more strategies (multifaceted intervention). Other strategies included: involvement in a stroke rehabilitation working party, Masters of Health Sciences course, and Stroke Collaborative.

**Figure 11:**
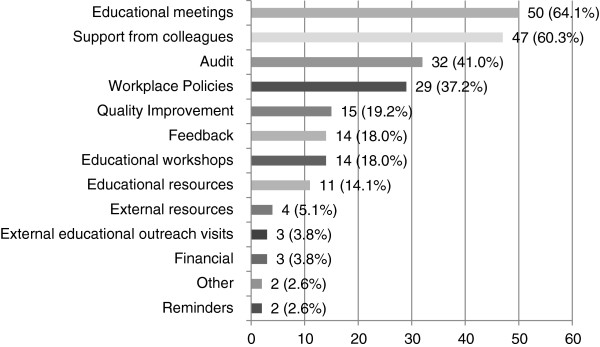
**The most useful strategies to help implement stroke CPGs after dissemination (n = 78).** Participants who received guideline implementation strategies were asked to indicate the top three most useful strategies.

Speech pathologists reported that the top three most useful strategies were educational meetings (64.1%), support from colleagues (60.3%), and audit (41.0%) (see Figure 11).

**Figure 12:**
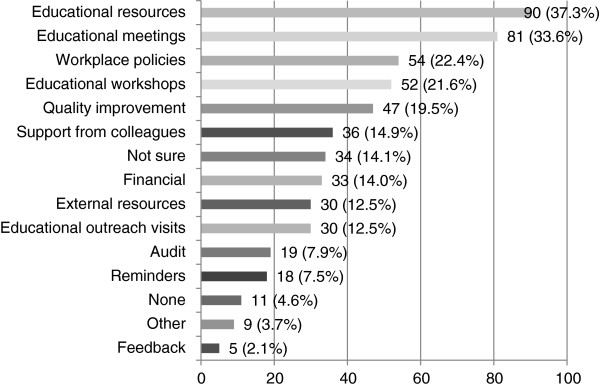
**Strategies that would have been most useful to implement stroke CPGs after dissemination (n = 241).** Participants who did and did not receive strategies were asked to indicate what three strategies would have been most useful to implement stroke CPGs.

Just over a third of all participants reported that educational resources (37.3%), educational meetings (33.6%), workplace policies (22.4%), and educational workshops (21.6%) would have been most the helpful if they could receive it (see Figure 12). Additional comments indicated that strategies such as making the guidelines a regular agenda item in team meetings, receiving support from management, more resources such as time and staff, and stronger research evidence-base would also support implementation.

### Other additional comments regarding stroke clinical practice guidelines (Q39)

Thirty-six respondents provided novel additional comments. These responses were grouped into three categories. The first category identified particular work environments that were barriers to implementation, but were not reported previously. Respondents reported that a rural geographical location and their role as a generalist clinician made it more difficult to implement stroke CPG recommendations. The second group of comments indicated that the guideline had important omissions including *“…no allowances for right hemisphere strokes or really low level dysphasics”* and perceived a lack of specificity for communication-related recommendations compared to dysphagia-related recommendations. Finally, speech pathologists also reported that they required greater practical assistance beyond general education to implement CPGs, for example, it is “*not particularly easy to pick up and run with. It would be good if more practical educational materials or protocols could be developed to assist clinicians”.*

## Discussion

This study described Australian speech pathologists’ experiences and use of stroke CPGs, and what influenced their ability to implement stroke CPG recommendations in clinical practice.

### Experiences and current use of stroke clinical practice guidelines

#### Experiences with stroke clinical practice guidelines

Speech pathologists in this study generally had a positive experience with stroke CPGs. Almost all speech pathologists were aware of stroke CPGs and had used them. More than 80% of participants reported that stroke CPGs had helped improve the care they provide due to reasons such as the guideline providing a standard they could trust, keeping them up to date with clinical stroke care research, and developing or improving services. There were only a few speech pathologists that had negative experiences with stroke CPGs. For example, 2.4% of respondents found that the guidelines were not very useful or not useful at all.

#### Current use of stroke clinical practice guidelines

Speech pathologists had predominately used stroke CPGs to implement the best available research evidence, improve clinical practice outcomes, and guide decision-making. More than half of the responses indicated that the CPGs had been used to develop internal policies, procedures, or pathways. The stroke CPG had also been used to advocate for clients and services such as the formation of a stroke unit.

There are differences between speech pathologists’ use of CPGs compared with other health professionals. The use of guidelines to implement the best available research, to develop policies, pathways, and procedures; and to advocate for clients and services had not been reported in studies on other health professionals such as nurses and physiotherapists [36-38].

A key finding was that for speech pathologists, stroke CPGs were used and had helped improve stroke care in a way that reflected the three types of knowledge use in the literature [39], that is; conceptually, instrumentally, and strategically. Firstly, the guidelines were used conceptually because it had changed their level of knowledge, such as increasing their up-to-date stroke care knowledge. Secondly, stroke CPGs had been used instrumentally by changing their behaviour or clinical practice, through embedding the guideline into local pathways or policies. Finally, the stroke CPG had been used strategically because the speech pathologist attained specific power or profit by using the guideline to advocate for patients and services.

### Factors that influenced speech pathologists to use stroke clinical practice guidelines

All broad areas identified in the literature that influenced the continued use of CPGs were demonstrated in the current study of speech pathologists, including the (a) guideline, (b) health professionals, (c) patient characteristics, (d) work environment, and (e) type of implementation strategy [17,22-24,26]. Consistent with previous research on occupational therapists [24], these categories could be both a facilitator and barrier to the use of stroke CPGs depending on the presence or absence of individual factors, or the context of the situation. For example, time was a barrier to the use of stroke CPGs when absent; but a facilitator when present. In another example, the stroke CPG’s brevity was helpful for some speech pathologists, whereas for others, the brevity was limiting. Additionally, as expected, there were factors influencing speech pathologists within these five categories, that were not reported by other health professionals in previous studies. This highlights the complex nature of factors that can influence guideline implementation and the importance of understanding local contexts and users.

#### (a). Factors within the stroke clinical practice guideline

The main barrier relating to the guideline itself that hindered the use of stroke CPGs was that the recommendations in stroke CPGs were felt to be impractical. This factor was not reported or examined in previous studies on allied health professionals [22-24], possibly due to the likelihood of an overlap in the attribution of whether this is an issue with guideline itself or limitations in some work environments (e.g. insufficient staffing).

The guideline itself facilitated stroke CPG implementation when the guideline: presented information clearly, promoted client centred care, and was based on evidence. These findings are consistent with other research that found that guidelines were easier to use when they presented information clearly [17,23] and were based on evidence [17]. Although the promotion of client centred care has not been examined in other studies on allied health professionals, it is consistent with the use of CPGs by nurses, as they are less likely to use CPGs when the guideline conflicts with family goals [40] or standardised care [41].

#### (b). Factors related to the speech pathologists themselves

Surprisingly, only a small percentage of participants had reported factors that related to themselves that hindered the use of stroke CPGs. Thus it seems that this category may not have a great influence in the implementation of CPGs by speech pathologists, compared to other health professionals [17]. Nevertheless, some barriers reported were insufficient skills to implement the guidelines and experiencing a tension between their own experience and the guideline recommendations. These barriers are consistent with previous qualitative and quantitative research on physicians, occupational therapists, and physiotherapists [17,22,23]. In contrast to the meta-review on mainly physicians [17], the present study found that there was a larger proportion of speech pathologists with less clinical experience who had not used stroke CPGs. This result was not expected and the reasons why participants with less clinical experience did not use stroke CPGs was not explored in this study. However, it is possible that less experienced speech pathologists relied on other tools such as colleagues, as research on new graduate occupational therapists indicated that they predominately relied on colleagues for clinical learning and knowledge [42].

A desire to implement evidence-based practice was by far the most frequent factor reported that related to the speech pathologist themselves and influenced them to use stroke CPGs (97.9%). This is consistent with a recent study that found that speech pathologists had the highest attitude towards evidence-based practice compared to occupational therapists, nutritionists/dieticians, social workers, and physiotherapists at baseline level [43].

#### (c). Factors relating to characteristics of the patient

The influence of patient characteristics on guideline use was not directly probed for in the survey. However, some speech pathologists commented on patient characteristics that influenced their use of stroke CPGs in open ended questions. This indicates that it can be a factor that influences speech pathologists’ use of stroke CPGs although more research on this factor is needed. Speech pathologists described that it was difficult to implement stroke CPG recommendations with patients who had multiple co-morbidities or severe impairments. For example, one speech pathologist reported that a patient with severe aphasia could not cope with the recommended treatment intensity in the guideline, thus complete compliance with the CPG in these circumstances was not appropriate.

#### (d). Factors within the work environment

The main barrier related to the work environment was a lack of time, education, treatment resources, and package or standardised assessments, to carry out guideline recommendations; which is universally consistent with the majority of research in this area [17,22-24,26]. Some speech pathologists also reported that working in a rural or remote setting hindered their use of stroke CPGs. This difficulty had also been reported by rural urologists [44], but has not been examined in other allied health professionals. A further four barriers in the work environment were identified in this study that had not been reported in the literature previously. Firstly, a significantly larger proportion of speech pathologists who worked in private practice did not use stroke CPGs. Private speech pathologists may receive less intensive support from a multidisciplinary team and other colleagues, and are under more pressure to account for their direct client contact billable time; and thus may find it difficult to implement stroke CPGs. Furthermore, speech pathologists working in a community setting, Geriatric Evaluation and Management (GEM) unit, or across multiple settings (e.g. acute inpatients, community, outpatients, rehabilitation ward, and nursing homes) found it difficult to use stroke CPGs due to limited time and resources; and a perceived lack of stroke recommendations tailored to this continuum of care.

In relation to the work environment, the main factors that facilitated the use of guidelines included support from supervisors or colleagues to use the guidelines; workplace policies, pathways or procedures; and working in a multidisciplinary team. These facilitators were also reported by health professionals such as physicians, nurses, and occupational therapists previously [18-20,22,26,45]. Teamwork is a significant factor that influences the successfulness of stroke rehabilitation training programs and hence patient outcomes, such as functional gains [46] and length of hospital stay [47]. Thus supporting and collaborating with the multidisciplinary team and other speech pathologists seems to be essential when improving guideline implementation.

#### (e). Implementation strategies

Only one third of speech pathologists reported that they were provided with strategies or support to help implement the stroke CPG after the guideline was disseminated. As the overwhelming evidence suggests that dissemination of CPG alone is ineffective [18,19], this may further illustrate why speech pathologists may find it difficult to implement guidelines.

The most frequent implementation strategies provided to speech pathologists were support from colleagues, educational meetings, and/or workplace policies. Although support from colleagues and educational meetings have been found to be effective in implementing guideline use [18,19], workplace policies have not been considered in previous studies [18-20]. Provision of a structural workplace policy or “rules” may be perceived as a guideline implementation strategy. Moreover, some strategies that have promising evidence behind them were not provided to speech pathologists, whilst other strategies that were provided have questionable evidence. For example, despite the extensive literature that reports that external educational outreach visits were effective in implementing guidelines [18-20], only four speech pathologists were provided with this strategy. Whereas 15 participants were given financial incentives despite the inconclusive nature on the effectiveness of this strategy [19].

Surprisingly, implementation strategies that were reported to be most effective for speech pathologists differed from results of systematic reviews on other health professionals. Speech pathologists indicated that educational meetings, educational resources, and support from colleagues were or would be the most useful strategy to assist with guideline implementation. This contrasts the extensive review on other health professionals where providing reminders was the most commonly reported effective strategy, followed by audit and feedback, opinion leaders, and educational outreach visits [18-20]. Speech pathologists in this study reported reminders to be the least effective strategy which was not expected.

### Understanding the factors that influence the implementation of stroke clinical practice guidelines in terms of the knowledge-to-action framework

The utilisation of the KTA framework in the survey design had illuminated other guideline implementation factors that go beyond a specific focus on barriers and facilitators and implementation strategies typically researched in the literature [17-24,26]. Therefore, a preliminary mapping exercise was conducted to conceptualise and further highlight the findings of the survey in relation to the KTA framework. These are presented in Table 3 and Table 4.

**Table 3:** Survey results mapped onto the knowledge creation component of the knowledge-to-action framework

**Knowledge creation component**	
Knowledge inquiry and synthesis	Participants recognised that a greater number of research studies are required and the lack of high level evidence in speech pathology can affect the degree of implementation e.g. “*I think not all available evidence has been incorporated into the guidelines”*.
Products/Tools	The stroke CPG had acted as a tool by helping speech pathologists implement evidence-based practice, improve patient outcomes, and guide decision-making. Aspects of the guideline that helped implementation included its clarity of information, level of evidence base, and ability to promote client centred care. However, the usability of the guideline is affected by limitations of the CPG such as impractical recommendations and insufficient information provided. The static nature of the tool meant that it could easily be out of date.

**Table 4:** Survey results mapped onto the action cycle component of the knowledge-to-action framework

**Action cycle component**	
Identifying a problem	Speech pathologists identified evidence to practice gaps and that audits provided assistance to identify and address those gaps.
	However, not all services were auditing their practice and respondents acknowledged some gaps went unaddressed.
Identifying, review, select knowledge	The majority of respondents were aware of stroke CPGs and had used the guidelines, with most utilising the 2010 National Stroke Foundation guideline. Most respondents reported that the stroke CPG were “somewhat useful” or “very useful”. 46 participants did not use the guidelines and the reasons for their non-use remains unknown.
	Some speech pathologists still acknowledged the need to continue to select, examine, and synthesise the broader and more recent literature. Participants also identified fields of evidence not sufficiently addressed in the guidelines e.g. right hemisphere stroke, severe aphasia, long-term stroke management.
Adapt the knowledge to local context	Over half of the participants had adapted the stroke CPG to their clinical setting in pathways, policies, or procedures. Others had not had the opportunity to implement the CPG in their local context.
Access barriers to knowledge use	Barriers and facilitators to the continued use of stroke CPGs were:
	(a) *The guideline itself*: e.g. facilitator: clarity of information; barrier: recommendations are not practical.
	(b) *Work environment (context/setting)*: e.g. facilitator: influence or interest from others; barrier: lack of time, staff, resources.
	(c) *Factors relating to the speech pathologist (adopters)*: e.g. facilitator: a desire to implement evidence-based practice; barrier: insufficient skills to implement the guideline. A greater proportion of clinicians who did not use stroke CPGs worked in private practice, did not work in a multidisciplinary team, or had 1-5 years experience working with neurogenic communication disorders.
	(d) *Patient characteristics*: e.g. patients with severe aphasia could be both a barrier and facilitator to the use of stroke CPGs depending on the context.
	(e) *Type of implementation strategy*: (See Selecting, tailoring ,and implement interventions below)
**Action cycle component**	
Selecting, tailoring, and implement interventions	Eighty (32.3%) of the 248 speech pathologists reported that they were provided with strategies or support to help implement the stroke CPG. Speech pathologists indicated that the most useful strategies are educational meetings, support from colleagues, auditing, and educational resources. All but one participant received multifaceted intervention.
Monitor knowledge use	250 respondents (84.6%) had used the stroke CPG in some way. The main reasons to use the guideline were to implement the best available research evidence, improve clinical practice outcomes, and to guide decision-making. The guidelines had also been used to inform clinical practice, develop pathways, and develop policies.
	The most common method to evaluate adherence to stroke CPGs were the National Stroke Foundation audit (45.3%), other workplace audits (34.9%), and use of quality indicators (32.8%). Seventy of 232 respondents reported that no evaluation took place of the implementation of stroke CPGs.
Evaluate outcomes	190 participants (80.5%) reported that the stroke CPG had helped improve the care they provided, and 46 (19.5%) indicated that it had not. The perceived reasons for how the guidelines have helped improve healthcare were:
	*Changed speech pathologist’s behaviour or knowledge*: e.g. something they could refer to, made them feel more confident in the services they provided, and encouraged them to read more literature.
	*Improved patient care*: e.g. more dysphagia screening within 24 hours, patients not being given inappropriate PEG tubes, less patients inappropriately being kept nil by mouth, less aspiration pneumonia.
	*Changed, developed, or improved workplace services*: e.g. advocate for services, encouraged client centred or multidisciplinary care, supported the development of policies and practices.
Sustain knowledge use	Speech pathologists identified strategies that helped them to continue to use the stroke CPG. For example, National Stroke Foundation audits and use of quality indicators. Obtaining detail data on the sustained use of stroke CPGs over a period of time was beyond the scope of this study.

As indicated in Tables 3 and 4, factors that could influence speech pathologists to implement guidelines can occur at any point during the search and collation of evidence and the development of the CPG (Knowledge Creation), as well as during the clinical process of implementing the guideline in practice (Action Cycle). For example, using the stroke CPG to develop pathways and policies demonstrated that speech pathologists had adapted and tailored the guidelines to their own local clinical setting. Adapting guidelines to the local work environment has been shown to improve CPG adherence [48] and is one component of the KTA framework. The evaluation of the implementation of guidelines in clinical practice is another component of the KTA framework. Some speech pathologists had used the National Stroke Foundation audit, other workplace audits, or quality indicators to evaluate whether stroke CPGs had been implemented. Auditing [49] and quality indicators [50] to evaluate the use of CPGs have been shown to improve guideline compliance. It is likely that a breakdown at any point in the KTA framework may prevent the successful implementation of guidelines in clinical practice.

Therefore, the KTA framework provides a useful and theoretically motivated way to conceptualise the many different factors that influence the implementation of research evidence into practice.

### Clinical implications

Speech pathologists should continue to use stroke CPGs in their workplace as it can help to improve the care provided for patients who have had a stroke, their organisation, and for themselves as a clinician. They should work together and also with the multidisciplinary team to improve stroke CPG implementation, for example, participate in team training programs. Speech pathologists may want to consider applying the KTA framework when implementing stroke CPGs in their workplace as these processes may influence guideline implementation. When identifying guideline implementation factors within the KTA framework, they should consider the local context and individual clinician. This may help illuminate the local factors that affect CPG adherence in individual settings. Speech pathologists could then develop tailored strategies to minimise or remove barriers and put in place facilitators to enhance guideline implementation, as indicated in Tables 3 and 4.

### Strengths, limitations, and future recommendations

This is the first study known to the authors to survey speech pathologists’ experiences with stroke CPGs; as well as the first to incorporate a theoretical model into the design of the survey. A major strength of this study is that a large diverse number of speech pathologists from different work environments, clinical experience, and demographic characteristics participated in the survey thereby increasing the external validity of the study. The literature review, reference group, and the KTA framework all enhanced the content and design of this survey, which has revealed many factors that have not been identified in previous literature and are relevant to speech pathologists who work in stroke care.

This study had several limitations. Whilst a survey methodology is useful in providing an overview of speech pathologists’ experiences with stroke CPGs, further research is needed to explore their experiences in more detail. Future studies could examine the effectiveness of tailored intervention versus untailored intervention on the use of stroke CPGs for speech pathologists. Further research is also needed to understand the factors that influence speech pathologists working in private, generalist, community, and rural and remote settings to implement guidelines. Research should also further explore the relationships between different variables in this study that may have influenced guideline implementation such as the influence of age, work environment, and years of clinical experience; on the use of stroke CPGs. Finally, future research could examine whether the KTA framework is a suitable model to help speech pathologists implement and continue to sustain the use of stroke CPGs.

## Conclusion

This study described speech pathologists’ use and experiences with stroke CPGs, and identified what enabled and hindered them to implement stroke CPG recommendations in clinical practice. This study revealed that there are numerous factors that can influence speech pathologists to implement the recommendations in stroke CPGs. The use of the KTA framework expanded the focus from traditional barriers and facilitators and guideline dissemination strategies to other aspects of implementation such as adapting the stroke CPG to the local work environment, monitoring the use of guidelines, evaluating the outcomes of using stroke CPGs, and sustaining the use of stroke CPGs.

## Competing interests

The authors declare that they have no competing interests.

## Authors’ contributions

We confirm that this manuscript has not been submitted in the present or any other form to any other journal for publication. This project was conceptualised by EP. KH carried out the literature review, research design, data collection, analysis and interpretation, and wrote and edited the manuscript. EP and RO supervised KH in the entire research process. EP and RO contributed input into all stages of the research study including write up and editing of the manuscript. All authors read and approved the final manuscript.

## Pre-publication history

The pre-publication history for this paper can be accessed here:

http://www.biomedcentral.com/1472-6963/14/110/prepub

## Supplementary Material

Additional file 1: Table S1 List of Survey Questions. Click here for file

## References

[B1] World Health Organization The Atlas of Heart Disease and Stroke 2004 Geneva: World Health Organization

[B2] Dickey L Kagan A Lindsay MP Fang J Rowland A Black S Incidence and profile of inpatient stroke-induced aphasia in Ontario, Canada Arch Phys Med Rehabil 2010 14 196 202 10.1016/j.apmr.2009.09.020 20159121

[B3] Vidović M Sinanović O Sabaskić L Haticić A Brkić E Incidence and types of speech disorders in stroke patients Acta Clin Croat 2011 14 491 493 22649878

[B4] Riepe MW Riss S Bittner D Huber R Screening for cognitive impairment in patients with acute stroke Dement Geriatr Cogn Disord 2004 14 49 53 10.1159/000074082 14560065

[B5] Martino R Foley N Bhogal S Diamant N Speechley M Teasell R Dysphagia after stroke: incidence, diagnosis, and pulmonary complications Stroke 2005 14 2756 2763 10.1161/01.STR.0000190056.76543.eb 16269630

[B6] Hilari K The impact of stroke: are people with aphasia different to those without? Disabil Rehabil 2011 14 211 218 10.3109/09638288.2010.508829 20712416

[B7] Altman KW Yu GP Schaefer SD Consequence of dysphagia in the hospitalized patient: impact on prognosis and hospital resources JAMA Otolaryngol Head Neck Surg 2010 14 784 789 10.1001/archoto.2010.129 20713754

[B8] McCurtin A Roddam H Evidence-based practice: SLTs under siege or opportunity for growth? The use and nature of research evidence in the profession Int J Lang Commun Disord 2012 14 11 26 10.1111/j.1460-6984.2011.00074.x 22268898

[B9] O’Connor S Pettigrew CM The barriers perceived to prevent the successful implementation of evidence-based practice by speech and language therapists Int J Lang Commun Disord 2009 14 1018 1035 19294555 10.1080/13682820802585967

[B10] Graham ID Logan J Harrison MB Straus SE Tetroe J Caswell W Robinson N Lost in knowledge translation: time for a map? J Contin Educ Health Prof 2006 14 13 24 10.1002/chp.47 16557505

[B11] Straus SE Tetroe J Graham I Defining knowledge translation CMAJ 2009 14 165 168 10.1503/cmaj.081229 19620273 PMC2717660

[B12] Institute of Medicine Clinical Practice Guidelines We Can Trust 2011 Washington, DC: The National Academies Press

[B13] Hubbard IJ Harris D Kilkenny MF Faux SG Pollack MR Cadilhac DA Adherence to clinical guidelines improves patient outcomes in Australian audit of stroke rehabilitation practice Arch Phys Med Rehabil 2012 14 965 971 10.1016/j.apmr.2012.01.011 22480546

[B14] National Stroke Audit Rehabilitation Services Report 2012 [http://www.strokefoundation.com.au/]

[B15] National Sentinel Stroke Audit 2010 Public Report [http://www.rcplondon.ac.uk]

[B16] National Report Stroke Services in Scottish Hospitals 2012 [http://www.strokeaudit.scot.nhs.uk]

[B17] Francke AL Smit MC de Veer AJE Mistiaen P Factors influencing the implementation of clinical guidelines for health care professionals: a systematic meta-review BMC Med Inform Decis Mak 2008 14 38 49 10.1186/1472-6947-8-38 18789150 PMC2551591

[B18] Grimshaw JM Thomas RE MacLennan G Fraser C Ramsay CR Vale L Whitty P Eccles MP Matowe L Shirran L Wensing M Dijkstra R Donaldson C Effectiveness and efficiency of guideline dissemination and implementation strategies Health Technol Assess 2004 14 iii-iv 1 72 10.3310/hta8060 14960256

[B19] Prior M Guerin M Grimmer-Somers K The effectiveness of clinical guideline implementation strategies: a synthesis of systematic review findings J Eval Clin Pract 2008 14 888 897 10.1111/j.1365-2753.2008.01014.x 19018923

[B20] Medves J Godfrey C Turner C Paterson M Harrison M MacKenzie L Durando P Systematic review of practice guideline dissemination and implementation strategies for healthcare teams and team-based practice Int J Evid Based Healthc 2010 14 79 89 20923511 10.1111/j.1744-1609.2010.00166.x

[B21] Hakkennes S Dodd K Guideline implementation in allied health professions: a systematic review of the literature BMJ Qual Saf 2008 14 296 300 10.1136/qshc.2007.023804 18678729

[B22] Bayley MT Harrison M Graham ID Hurdowar A Richards CL Korner-Bitensky N Wood-Dauphinee S Eng JJ McKay-Lyons M Harrison E Teasell R Barriers to implementation of stroke rehabilitation evidence: findings from a multi-site pilot project Disabil Rehabil 2012 14 1633 1638 10.3109/09638288.2012.656790 22631218

[B23] Otterman NM van der Wees PJ Bernhardt J Kwakkel G Physical therapists’ guideline adherence on early mobilization and intensity of practice at dutch acute stroke units: a country-wide survey Stroke 2012 14 2395 2401 10.1161/STROKEAHA.112.660092 22773556

[B24] Poitras S Durand MJ Côté AM Tousignant M Use of low-back pain guidelines by occupational therapists: a qualitative study of barriers and facilitators Work 2011 14 465 475 21811035 10.3233/WOR-2011-1196

[B25] Poitras S Durand M Côté A Tousignant M Guidelines on low back pain disability: interprofessional comparison of use between general practitioners, occupational therapists, and physiotherapists Spine (Phila Pa 1976) 2012 14 1252 1259 10.1097/BRS.0b013e31824b6adf 22310094

[B26] Verweij LM Proper KI Leffelaar ER Weel ANH Nauta AP Hulshof CTJ van Mechelen W Barriers and facilitators to implementation of an occupational health guideline aimed at preventing weight gain among employees in the Netherlands J Occup Environ Med 2012 14 954 960 10.1097/JOM.0b013e3182511c9f 22850353

[B27] SurveyMonkey [http://www.surveymonkey.com]

[B28] Kajermo K Boström A-M Thompson DS Hutchinson AM Estabrooks CA Wallin L The BARRIERS scale–the barriers to research utilization scale: a systematic review Implement Sci 2010 14 32 54 10.1186/1748-5908-5-32 20420696 PMC2883534

[B29] Richardson J Design and conduct a survey Complement Ther Med 2005 14 47 53 10.1016/j.ctim.2004.12.005 15907678

[B30] Braithwaite D Emery J De Lusignan S Sutton S Using the internet to conduct surveys of health professionals: a valid alternative? Fam Pract 2003 14 545 551 10.1093/fampra/cmg509 14507796

[B31] WINPEPI [http://www.brixtonhealth.com/pepi4windows.html]

[B32] General Membership Survey [http://www.speechpathologyaustralia.org.au]

[B33] Elo S Kyngäs H The qualitative content analysis process J Adv Nurs 2008 14 107 115 10.1111/j.1365-2648.2007.04569.x 18352969

[B34] Hsieh H Shannon SE Three approaches to qualitative content analysis Qual Health Res 2005 14 1277 1288 10.1177/1049732305276687 16204405

[B35] Krippendorff K Reliability in content analysis: some common misconceptions and recommendations Hum Comm Res 2004 14 411 433

[B36] Côté AM Durand MJ Tousignant M Poitras S Physiotherapists and use of low back pain guidelines: a qualitative study of the barriers and facilitators J Occup Rehabil 2009 14 94 105 10.1007/s10926-009-9167-2 19219536

[B37] Hendrick P Mani R Bishop A Milosavljevic S Schneiders AG Therapist knowledge, adherence and use of low back pain guidelines to inform clinical decisions: a national survey of manipulative and sports physiotherapists in New Zealand Man Ther 2013 14 136 142 10.1016/j.math.2012.09.002 23047043

[B38] Bahtsevani C Willman A Stoltz P Östman M Experiences of the implementation of clinical practice guidelines: interviews with nurse managers and nurses in hospital care Scand J Caring Sci 2010 14 514 522 10.1111/j.1471-6712.2009.00743.x 20070594

[B39] Huberman M Linking the practitioner and researcher communities for school improvement Sch Eff Sch Improv 1993 14 1 16 10.1080/0924345930040101

[B40] Colón-Emeric CS Lekan D Utley-Smith Q Ammarell N Bailey D Corazzini K Piven ML Anderson RA Barriers to and facilitators of clinical practice guideline use in nursing homes J Am Geriatr Soc 2007 14 1404 1409 10.1111/j.1532-5415.2007.01297.x 17767682 PMC2276683

[B41] Forsner T Hansson J Brommels M Wistedt AT Forsell Y Implementing clinical guidelines in psychiatry: a qualitative study of perceived facilitators and barriers BMC Psychiatry 2010 14 8 18 10.1186/1471-244X-10-8 20089141 PMC2822755

[B42] Toal-Sullivan D New graduates’ experiences of learning to practise occcupational therapy Br J Occup Ther 2006 14 513 513

[B43] Lizarondo LM Grimmer-Somers K Kumar S Crockett A Does journal club membership improve research evidence uptake in different allied health disciplines: a pre-post study BMC Res Notes 2012 14 558 597 10.1186/1756-0500-5-558 23106851 PMC3517354

[B44] Strope SA Elliott SP Saigal CS Smith A Wilt TJ Wei JT Urologist compliance with AUA best practice guidelines for benign prostatic hyperplasia in medicare population Urology 2011 14 3 9 10.1016/j.urology.2010.12.087 21601254 PMC3126893

[B45] Hill K Middleton S O’Brien E Lalor E Implementing clinical guidelines for acute stroke management: do nurses have a lead role? Aust J Adv Nurs 2009 14 53 58

[B46] Strasser DC Falconer JA Stevens AB Uomoto JM Herrin J Bowen SE Burridge AB Team training and stroke rehabilitation outcomes: a cluster randomized trial Arch Phys Med Rehabil 2008 14 10 15 10.1016/j.apmr.2007.08.127 18164324

[B47] Schouten LMT Hulscher MEJL Akkermans R van Everdingen JJE Grol RPTM Huijsman R Factors that influence the stroke care team’s effectiveness in reducing the length of hospital stay Stroke 2008 14 2515 2521 10.1161/STROKEAHA.107.510537 18617664

[B48] Fervers B Burgers JS Haugh MC Latreille J Mlika-Cabanne N Paquet L Coulombe M Poirier M Burnand B Adaptation of clinical guidelines: literature review and proposition for a framework and procedure Int J Qual Health Care 2006 14 167 176 10.1093/intqhc/mzi108 16766601

[B49] Ivers N Oxman AD Jamtvedt G Flottorp S Young JM Odgaard-Jensen J French SD O’Brien MA Johansen M Grimshaw J Audit and feedback: effects on professional practice and healthcare outcomes Cochrane Database Syst Rev 2012 14 CD000259 22696318 10.1002/14651858.CD000259.pub3 PMC11338587

[B50] van der Geest LGM Krijnen P Wouters MWJM Erkelens WGW Marinelli AWKS Nortier HJWR Tollenaar RAEM Struikmans H Improved guideline compliance after a 3-year audit of multidisciplinary colorectal cancer care in the western part of the Netherlands J Surg Oncol 2012 14 1 9 10.1002/jso.23038 22234959

