# Supplementary material for: Speech pathologists’ experiences with stroke clinical practice guidelines and the barriers and facilitators influencing their use: a national descriptive study
Source: BMC Health Serv Res. 2014 Mar 6;14:110. doi: 10.1186/1472-6963-14-110 (PMC4015602; doi:10.1186/1472-6963-14-110)
Supplement: Additional file 1: Table S1 — List of Survey Questions. [file 1472-6963-14-110-S1.doc]

**Additional file**

**Table 1. List of Survey Questions**

| Questions | |
| --- | --- |
| 1. | I am a speech pathologist who has seen at least one patient with a stroke, in the last 5 years. |
| 2. | The age range I fall into is: |
| 3. | My gender is: |
| 4. | The number of years since I graduated is: |
| 5. | My highest level of academic achievement is: |
| 6. | My combined years of experience working with neurogenic communication disorders is: |
| 7. | The approximate percentage of my caseload that contains people who have had a stroke is: |
| 8. | I predominantly work in an (tick one): |
| 9. | My current most predominant work environment is: |
| 10. | My current most predominant work environment is in the state/territory of: |
| 11. | My current most predominant work region is: |
| 12. | Do you work in a multidisciplinary team?  A multidisciplinary team is defined here as a team of health professionals such as medical, nursing, and allied health (including Occupational Therapist, Physiotherapist, Speech Pathologist, Social Worker, and Dietitian) |
| 13. | Are you a member of a dedicated Stroke Unit team?  A dedicated stroke unit team is defined as a team that meets all the four following criteria:  1. Co-located beds within a geographically defined unit  2. A dedicated team with members who have a special interest in stroke and/or rehabilitation. The minimum team would consist of medical, nursing and allied health (including Occupational Therapist, Physiotherapist, Speech Pathologist, Social Worker & Dietician)  3. The team meets at least once per week to discuss patient care  4. Regular programs of staff education and training relating to stroke (e.g. dedicated stroke inservice program and/or access to annual national or regional stroke conference) |
| 14. | Have you heard of "stroke clinical practice guidelines"? |
| 15. | Have you used stroke clinical practice guidelines in any way?  *e.g. downloaded it, read it, used it to help develop pathways/policies, used it to inform clinical practice* |
| 16. | How did you use the stroke clinical practice guideline(s)?  You may tick more than one answer |
| 17. | Which of the following stroke clinical practice guideline(s) have you used?  You may tick more than one answer |
| 18. | My overall impression when using stroke clinical practice guidelines is that they are: |
| 19. | How did you become aware of the stroke clinical practice guideline?  You may tick more than one answer |
| 20. | After receiving the guideline, were strategies or support provided to help you implement it?  *e.g. training sessions, education by colleague/opinion leaders* |
| 21. | Please indicate the strategies provided by ticking 'Yes' or 'No' |
| 22. | From your response above, what were the top three most useful strategies? Pick up to three.  Write down the letter(s) of your top three most useful strategies into the boxes below  *e.g. Audit = D, Workplace policies = G* |
| 23. | Are there any strategies that you didn’t receive, that would have been helpful in assisting you to use the guideline?  Please tick up to three. |
| 24. | Are there aspects of the stroke clinical practice guideline itself that has helped you to continue to use it? *(e.g. clarity of information, level of evidence provided, format/design of the guideline)* |
| 25. | Please specify the aspects of the guideline that helped you to continue to use it: |
| 26. | Has the work environment helped you to continue to use the stroke clinical practice guideline? *(e.g. audit, workplace policies, quality improvement, education, and training activities)* |
| 27. | Please specify how the work environment helped you to continue to use the guideline |
| 28. | Have any of the following helped motivate you to use the stroke clinical practice guideline? |

**Table 1 (continued). List of Survey Questions**

| Questions | |
| --- | --- |
| 29. | Do you think the stroke clinical practice guideline itself creates barriers to its use?  *(e.g. recommendations are not practical, hinders client centred care, information is not clear)* |
| 30. | Please specify how the guideline itself created barriers to its use |
| 31. | In your opinion, does the work environment create barriers to your use of the stroke clinical practice guideline?  *(e.g. insufficient time, workplace policies, lack of resources to carry out the recommendations)* |
| 32. | Please specify how the work environment created barriers to your use of the guideline |
| 33. | In your opinion, have any of the following made you feel reluctant to use the stroke clinical practice guideline? |
| 34. | In your opinion, have stroke clinical practice guidelines helped improve the care you provide? *(e.g. improve clinical practice outcomes)* |
| 35. | Why haven't stroke clinical practice guidelines helped improve the care you provide? |
| 36. | How have stroke clinical practice guidelines helped improve the care you provide? |
| 37. | In your opinion, the main purposes/reasons you used stroke clinical practice guidelines were to: |
| 38. | In your workplace, what strategies are used to evaluate the implementation of stroke clinical practice guidelines? |
| 39. | Is there anything else you want to say about stroke clinical practice guidelines? |
